# Intense Pituitary 18F-Fluorodeoxyglucose Positron Emission Tomography Uptake in a Patient with Diabetes Insipidus

**DOI:** 10.4274/balkanmedj.galenos.2019.2019.4.3

**Published:** 2019-07-11

**Authors:** Çiğdem Soydal, Demet Nak, Mine Araz, Nurdan Taçyıldız, Nuriye Özlem Küçük

**Affiliations:** 1 Department of Nuclear Medicine, Ankara University School of Medicine, Ankara, Turkey; 2 Department of Pediatric Oncology, Ankara University School of Medicine, Ankara, Turkey

This case discusses a 6-year-old male patient with a two-month history of polyuria and polydipsia (3800-4000 cc/d). The patient's 24-hour urine osmolality was 79 mOsm/kg, and it increased to 98 mOsm/kg; he lost 5.2% of his weight after 7-hour water deprivation test. The urine osmolality increased from 98 to 472 mOsm/kg at the 4^th^ hour of 10 mcg desmopressin test. Moreover, his renal functions were normal, his polyuria and polydipsia resolved on treatment with 60 μg desmopressin twice daily, and his electrolyte levels were in the normal range. The patient was diagnosed with central diabetes insipidus. Pituitary profile was in a reasonable limit, except for prolactin, which was 32 ng/dL that was slightly higher than the upper limit. Gadolinium-enhanced magnetic resonance imaging of the pituitary showed contrast-enhanced lesions in the brainstem, prominently in the pons, with diffusely increased intensity on T2 sequence and limited diffusion. Lesions had right cerebellar and left cerebral extensions. Magnetic resonance imaging also revealed heterogenous pituitary gland, thickened stalk, and loss of a hot signal (T1 weighted) of the posterior pituitary, which was suggestive of Langerhans cell histiocytosis. Cerebral fluid sampling and pituitary stalk biopsy were considered deferred. Maximum intensity projection 18F-fluorodeoxyglucose positron emission tomography (Figure 1a) images of the patient revealed pathological uptakes in cranium, thorax, and the left pelvic region. Fused trans-axial images demonstrated pathological uptakes located in the pituitary gland (SUV_max_: 9.8) (Figure 1b), soft tissues in the anterior mediastinum (SUV_max_: 10.7) (Figure 1c), and the left acetabular region (SUV_max_: 10.6) (Figure 1d). Written informed consent was obtained from the parents.

The 18F-fluorodeoxyglucose uptake by the pituitary gland in patients with central diabetes insipidus related to different primary pathologies has been reported in earlier studies (2,5,6,7). Central diabetes insipidus has distinct etiologies in accordance with age (1,3,6). Diagnosis of central diabetes insipidus in children is straightforward when central diabetes insipidus follows a recent history of meningitis, neurosurgery, trauma, or known disease like Langerhans cell histiocytosis (3,6). Langerhans cell histiocytosis, with its central nervous system involvement, is the most common systemic disease that causes central diabetes insipidus in children (4,5). Central diabetes insipidus might be the first sign of so far undiagnosed extracranial disease, and an 18F-fluorodeoxyglucose positron emission tomography/computed tomography has an excellent potential to detect extracranial symptoms of these conditions (7). In this case, we aimed to share our experience of the intense 18F-fluorodeoxyglucose uptake by the pituitary gland in patients with central diabetes insipidus related with Langerhans cell histiocytosis involvement.

## Figures and Tables

**Figure 1:**
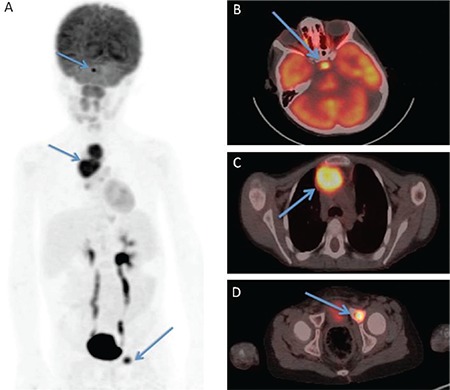
Maximum intensity projection 18F-fluorodeoxyglucose positron emission tomography images of the patient revealed pathological uptake in the cranium, thorax, and left pelvic region (a). Fused trans-axial images demonstrated pathological uptakes, located in the pituitary gland (SUV_max_: 9.8) (b), soft tissues in the anterior mediastinum (SUV_max_: 10.7) (c), and the left acetabular region (SUV_max_: 10.6) (d).
